# Supplementary material for: A high-resolution mRNA expression time course of embryonic development in zebrafish
Source: eLife. 2017 Nov 16;6:e30860. doi: 10.7554/eLife.30860 (PMC5690287; doi:10.7554/eLife.30860)
Supplement: Supplementary file 6. [file elife-30860-supp6.zip › biolayout-clusters-files/Cluster169-genes.html]

Cluster169


# Cluster169: Genes

| Ensembl ID | Gene Name | Chr | Start | End | Biotype |
| --- | --- | --- | --- | --- | --- |
| ENSDARG00000038359 | enosf1 | 7 | 56807803 | 56828567 | protein\_coding |
| ENSDARG00000062501 | hhipl1 | 17 | 17919259 | 17928751 | protein\_coding |
| ENSDARG00000009939 | micu2 | 5 | 35998954 | 36009547 | protein\_coding |
| ENSDARG00000100731 | slc27a2b | 25 | 31861929 | 31900099 | protein\_coding |
| ENSDARG00000056239 | tmem45b | 21 | 25199692 | 25210447 | protein\_coding |
| ENSDARG00000037455 | ugt8 | 1 | 19863130 | 19900841 | protein\_coding |
| ENSDARG00000011521 | upb1 | 8 | 30690197 | 30807715 | protein\_coding |
| ENSDARG00000074809 | zgc:194392 | 17 | 30683664 | 30685448 | protein\_coding |
